# Diffusion tensor microscopy data (15.6 μm in-plane**)** of white matter tracts in the human, pig, and rat spinal cord with corresponding tissue histology

**DOI:** 10.1016/j.dib.2016.08.020

**Published:** 2016-08-18

**Authors:** Jeremy J. Flint, Brian Hansen, Stephen J. Blackband

**Affiliations:** a Department of Neuroscience, University of Florida, Gainesville, FL, USA; b McKnight Brain Institute, University of Florida, Gainesville, FL, USA; c Center for Functionally Integrative Neuroscience, Aarhus University, Denmark; d Center for Structural Biology, University of Florida, Gainesville, FL, USA; e National High Magnetic Field Laboratory, Tallahassee, FL, USA

**Keywords:** Magnetic Resonance Imaging (MRI), Magnetic Resonance Microscopy (MRM), Diffusion Tensor Imaging (DTI), Tractography, Spinal Cord, Myelin, α-Motor Neurons

## Abstract

The following article contains nine diffusion tensor imaging (DTI) datasets acquired with magnetic resonance microscopy (MRM, 15.6 μm in-plane). All data was collected in the region bordering the ventral horn and white matter of cross sections from the spinal cord enlargements along with each sample׳s corresponding tissue histology. These data are collected in fixed spinal cord sections of varying thicknesses taken from rat (2×21 direction DTI datasets), pig (1×21 direction DTI dataset), and human (5×21 direction DTI datasets + 1×6 direction DTI dataset) tissue sources. Following MRM acquisition, the sections were histologically processed using Nissl or Black-Gold II (Histo-Chem Inc., 1BGII) myelin stain and imaged again using light microscopy techniques. Methodological procedures are an amalgamation of protocol components described previously (doi:10.1016/j.neuroimage.2010.04.031 [1], doi:10.1016/j.neuroimage.2011.04.052 [2]).

**Specifications Table**

**Table:** 

Subject area	*Structural Biology*
More specific subject area	*Cellular-Resolution MRI*
Type of data	*Figures, .MAT files*
How data was acquired	*MRM Data: Oxford Magnet (600* *MHz) interfaced to a Bruker Avance II spectrometer console running ParaVision 4.0 software*
	*Light Microscopy Data: 100x and 200x magnification images taken with a* Zeiss Axioplan 2 *microscope interfaced to a QImaging, Retiga 4000* *R Fast 1394 Color digital camera running QCapture Pro 6.0 software.*
Data format	*Raw*
Experimental factors	*Tissue slices fixed with 4% formaldehyde in a phosphor-buffered saline (PBS) solution prior to MRI. These same samples were then stained with Nissl or Black-Gold II prior to light microscopy.*
Experimental features	*DTI data was collected using an Ultra-High Field Strength magnet system (600* *MHz / 14.1* *T) and specialized micro radio-frequency surface coils. This equipment allows MR microscopy and histology to be performed on the same tissue samples, permitting a one-to-one comparison of both types of imaging data at equivalent resolution*
Data source location	*Gainesville, FL. USA 29.6516 −82.3248*
Data accessibility	*Data is provided with the article and stored in the Mendeley Data repository.*
	doi:10.17632/h3wkngnptj.1
	https://data.mendeley.com/datasets/h3wkngnptj/draft?a=4cff43de-7860-4f97-9342-2c3bdc91b51b

**Value of the data**

• Collected using an Ultra-High Field Strength magnet system (600 MHz / 14.1 T) and specialized micro radio-frequency coils which increases access to data generated with scarce hardware.
• Protracted MR scan intervals (16 h 25 min – 49 h per dataset) necessary for experiments means a significant cost and time savings to groups who are able to use our existing data.
• MR microscopy and histology are performed on the same tissue samples, meaning a one-to-one comparison of both types of imaging data at equivalent resolution becomes possible.
• This equivalence allows tractographic algorithms to be tested on the MRM tensor datasets and compared to the “ground truth” white matter structure visualized in the histology data.

## 1 Data

For these nine collections, diffusion tensor MRM data (15.6 μm in-plane) were acquired on fixed, excised cross sections of spinal cord isolated from human, pig, and rat samples [1], [2]. Eight of the datasets were 21-direction DTI acquisitions, while the last was a six-direction DTI data set collected to test the functional limits of minimum tensor acquisitions. MRM datasets are provided as matlab files (.mat). Each contains one image plane per sample as a 3D matrix: (X dimension, Y dimension, diffusion weighting [*b*=0 + 6 or 21 directions at *b*>0], the corresponding effective *b*-values, effective *b*-matrix [3×3,7 or 22], *b*-value diffusion cross-term contributions, *b*-value diffusion gradient contribution, *b*-value imaging gradient contributions). With this information, the data can be exported from matlab to any processing toolbox in the user׳s preferred data format (dicom, nifti etc). Each MRM dataset is paired with its corresponding Nissl or Black-Gold II stained histology image such that the MR data—taken at the tissue boundary between gray and white matter—is representative of the fraction of tissue in the histology image excited by the RF surface coil.

## 2 Experimental design, materials and methods

### 2.1 Tissue acquisition and processing

Human tissue samples were processed by and procured from Science Care, Inc. This tissue was immersion fixed in 10% formalin prior to shipment and stored after arrival in 4% formaldehyde dissolved in PBS solution (137 mM NaCl, 10 mM Na_2_HPO_4_, 2.7 mM KCl and 1.8 mM KH_2_PO_4_: pH = 7.3–7.4). Excised spinal cord enlargements from pig were immersion fixed and stored in the 4% formaldehyde PBS solution. Rats underwent exsanguination with injectable, 0.9% saline solution followed by cardiac perfusion using the same 4% formaldehyde solution used for the human and pig samples. All human and animal procedures were reviewed and approved by the institutional review board (IRB) and institutional animal care and use committee (IACUC) of the University of Florida respectively. Prior to imaging, samples were affixed to a cutting chuck using cyanoacrylate adhesive and sliced in PBS (4 ° C, pH 7.3–7.4; 300 mOsm). Serial cross sections (100 μm in pig, 25 μm in rat, and 50 μm in human) of spinal cord were cut with a Lancer 1000 vibratome (Ted Pella). The slices were washed in a PBS bath at ambient temperature overnight in order to reduce the levels of formaldehyde: the presence of which has been shown to affect T1 and T2 relaxation rates and estimates of diffusion in MR data [3].

### 2.2 Diffusion tensor imaging

Imaging studies were performed on a 600 MHz Oxford magnet and Avance II Bruker imaging spectrometer interfaced with a 500 μm diameter, four-turn micro surface coil (B6370) designed and fabricated by Bruker Instruments Inc. [4], [5]. For each dataset, an individual slice was chosen that featured cell bodies of α-motor neurons in close proximity to the edge of the ventral horn. This way, all structures of interest could be made to fit within the miniscule excitation profile of the micro surface coil. Proper placement of the samples such that these anatomical landmarks were overlapping the coil face was accomplished by hand using blunt-ended glass probes (300–500 μm tip) and a dissecting scope (Zeiss, OPMI 1-FC). Nylon components consisting of a circular mesh cutout (50 μm pore size) and notched retention ring were used to prevent the samples from moving and ensure they remained in constant contact with the surface coil face during imaging [6]. Once the sample was secured, PBS solution was used to fill the tissue well prior to sealing with PCR film (12 mm^2^, AB-0558). For the human samples, five 21-direction and one 6-direction diffusion tensor imaging datasets were collected (TR/TE=2000/17.5 ms, *b*=2000 s/mm^2^, Δ=8.36 ms, *δ*=2.0 ms, matrix=128×128, in-plane res=15.6 μm, Avg=15/scan, time=23.5 h). In the rat tissue, two 21-direction tensor datasets were collected (TR/TE=500/36 ms, Δ=17.0 ms, *δ*=6.0 ms, matrix=128×128, in-plane res=15.6 μm, *b*=3750 s/mm^2^, avg at *b*(0)=60, avg at *b*(3750)=30, time=49 h). For the pig sample, one 21-direction tensor dataset was collected (TR/TE=500/36 ms, Δ=17.0 ms, *δ*=6.0 ms, matrix=128×128, in-plane res=15.6 μm, *b*=3750 s/mm^2^, avg at *b*(0)=20, avg at *b*(1800)=10, time=16.5 h).

### 2.3 Histology and light microscopy

After MRM collection, samples were removed from the magnet and stained using either Nissl or Black-Gold II (Histo-Chem Inc., 1BGII). For Nissl staining, samples were placed in a staining bath (0.5% cresyl violet, 0.3% ethanoic acid, fill deionized water) for approximately 4 min followed by immersion in destain (0.3% ethanoic acid in deionized water) for 1 min. These slices were then stored briefly in PBS. In the case of Black-Gold II myelin stain, samples were incubated at 60 °C in a staining bath (0.3% dye, 0.9% NaCl, fill deionized water) for 12 min followed by immersion in a sodium thiosulfate stop-bath (1% Na_2_S_2_O_3_ in deionized water) at ambient temperature for 3 min. Stained samples were wet-mounted and imaged at 100× magnification (human and pig) or 200× magnification (rat). Histology was conducted on an Axioplan 2 microscope (Zeiss) interfaced to a Retiga 4000R color camera (QImaging). The resulting images were processed using QCapture Pro version 6.0 imaging software (QCapture). Black and white (B&W) as well as true-color (RGB) images were taken using the camera and software settings designated for these collections. False-color spectra were generated by manual manipulation of the images’ color (RGB) histograms as a means of improving contrast between Nissl-stained tissue components.

## References

[1] Flint J.J., Hansen B., Fe M., Schmidig D., King M.A., Vestergaard-Poulsen P., Blackband S.J. (2010). Cellular-level diffusion tensor microscopy and fiber tracking in mammalian nervous tissue with direct histological correlation. NeuroImage.

[2] Hansen B., Flint J.J., Heon-Lee C., Fey M., Schmidig D., King M.A., Vestergaard-Poulsen P., Blackband S.J. (2011). Diffusion tensor microscopy in human nervous tissue with quantitative correlation based on direct histological comparison. NeuroImage.

[3] Shepherd T.M., Thelwall P.E., Stanisz G.J., Blackband S.J. (2009). Aldehyde fixative solutions alter the water relaxation and diffusion properties of nervous tissue. Magn. Reson. Med..

[4] Massic C., Boreo G., Vincent F., Abenhaim J., Besse P.-A., Popovic R.S. (2002). High-Q factor RF planar microcoils for micro scale NMR spectroscopy. Sens. Actuators A.

[5] Weiger M., Schmidig D., Denoth S., Massin C., Vincent F., Schnekel M., Fey M. (2008). NMR microscopy with isotropic resolution of 3.0 μm using dedicated hardware and optimized methods. Concepts Magn. Reson. Part B.

[6] Flint J.J., Menon K., Hansen B., Forder J., Blackband S.J. (2015). A microperfusion and in-bore oxygenator system designed for magnetic resonance microscopy studies on living tissue explants. Sci. Rep..

