# Supplementary figures and images for: Diffusion tensor microscopy data (15.6 μm in-plane) of white matter tracts in the human, pig, and rat spinal cord with corresponding tissue histology
Source: Data Brief. 2016 Aug 18;9:271–4. doi: 10.1016/j.dib.2016.08.020 (PMC5024313; doi:10.1016/j.dib.2016.08.020)

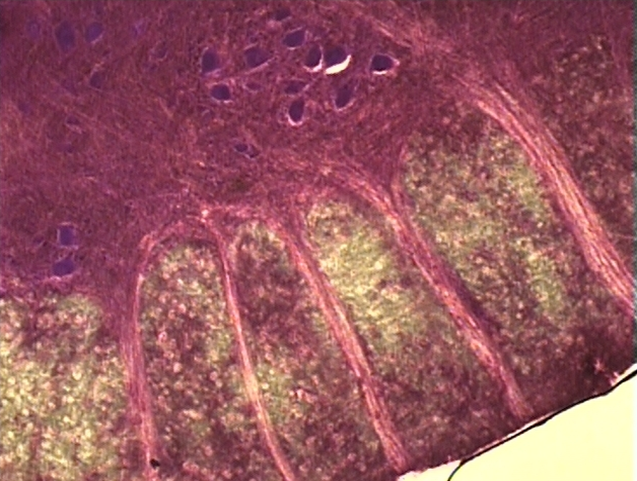

Supplement: Supplementary file 2 — Supplementary material [file mmc2.zip › data_sorted/pig_sc/set1/Histology/Pig111308_scans 7 to 28_Nissl stain_100um thick_true color.tif]

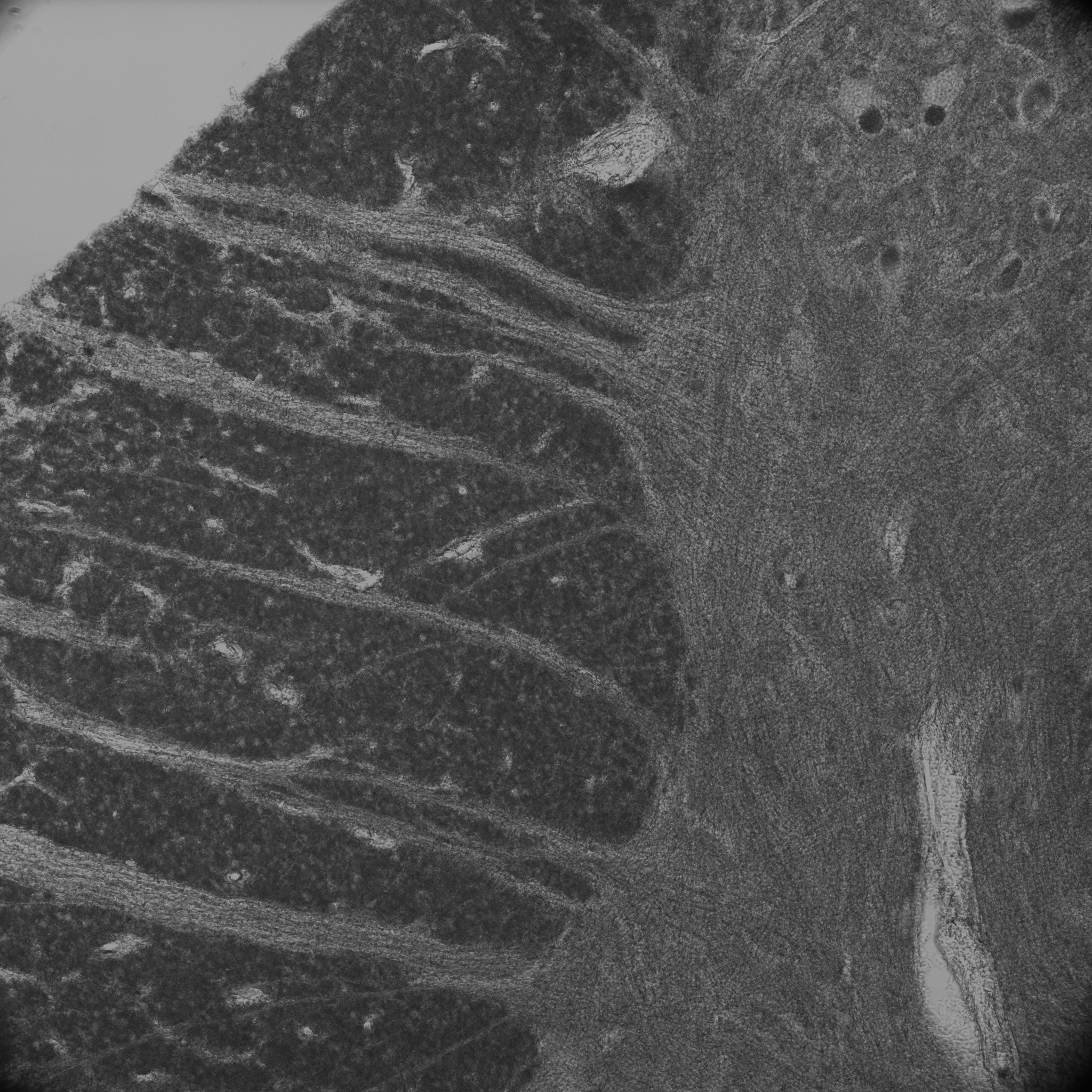

Supplement: Supplementary file 2 — Supplementary material [file mmc2.zip › data_sorted/human_sc/humsc_bg_set1/Histology/Hum051110_scan208_229_human SC_BlackGoldII_50um slice_10XB&W_2.tif]

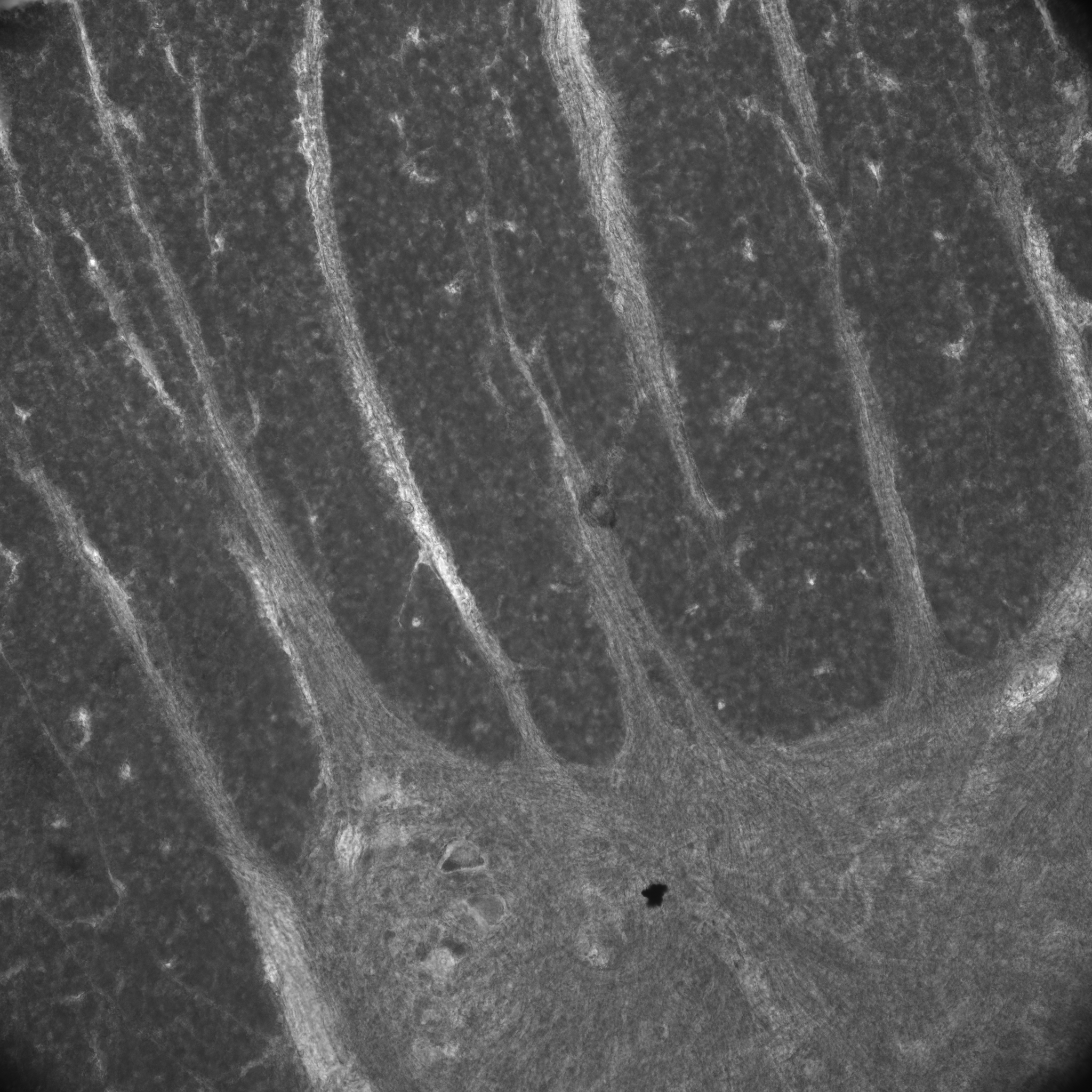

Supplement: Supplementary file 2 — Supplementary material [file mmc2.zip › data_sorted/human_sc/humsc_bg_set2/Histology/Hum051110_scan240-261_human SC_BlackGoldII_50um slice_10XB&W_2.tif]

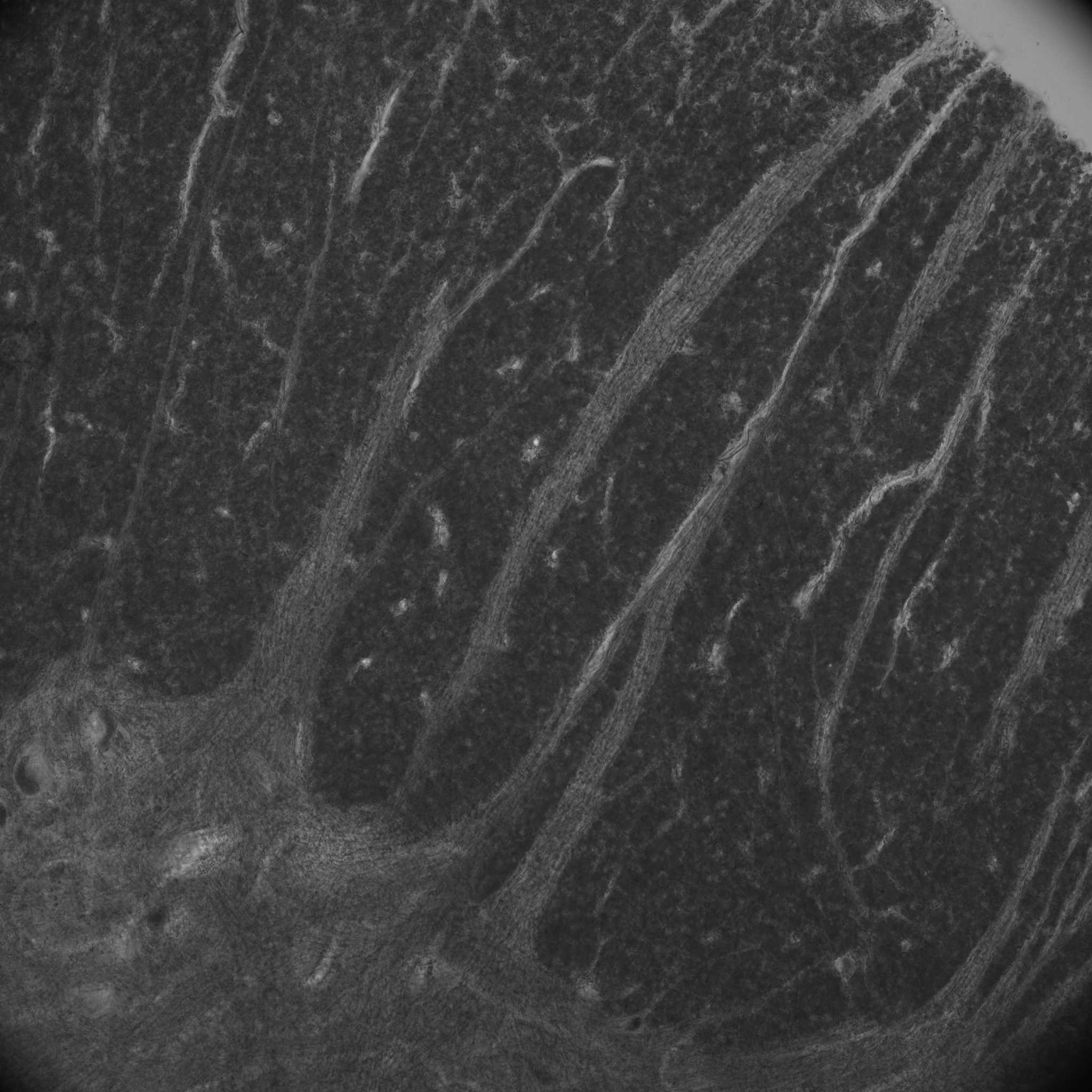

Supplement: Supplementary file 2 — Supplementary material [file mmc2.zip › data_sorted/human_sc/humsc_bg_set3/Histology/Hum051110_scan273-294_human SC_BlackGoldII_50um slice_10XB&W_2.tif]

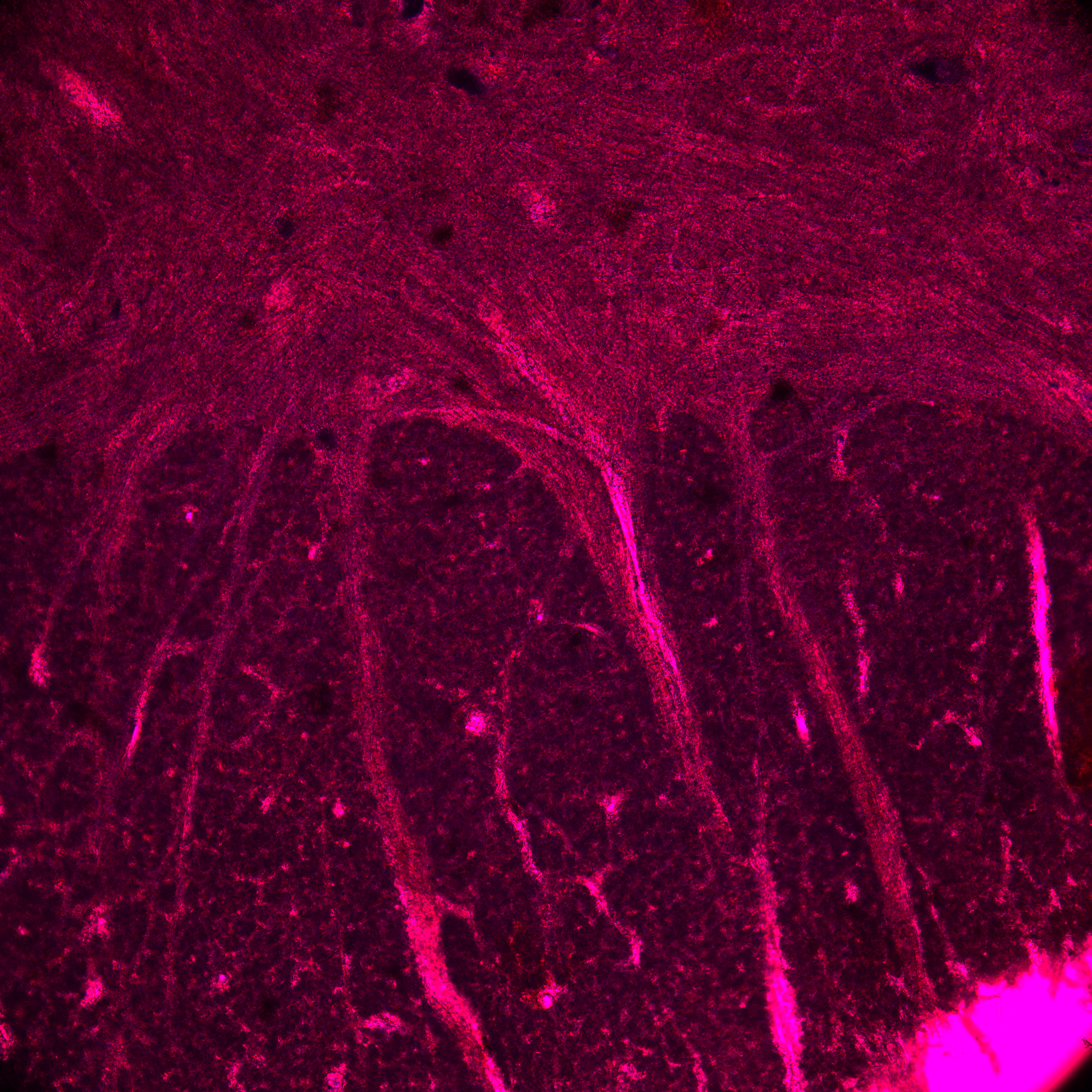

Supplement: Supplementary file 2 — Supplementary material [file mmc2.zip › data_sorted/human_sc/set1/Histology/Hum071409_HumanDTI_scans35-56_100X4_Nissl stained_50um slice_false color.tif]

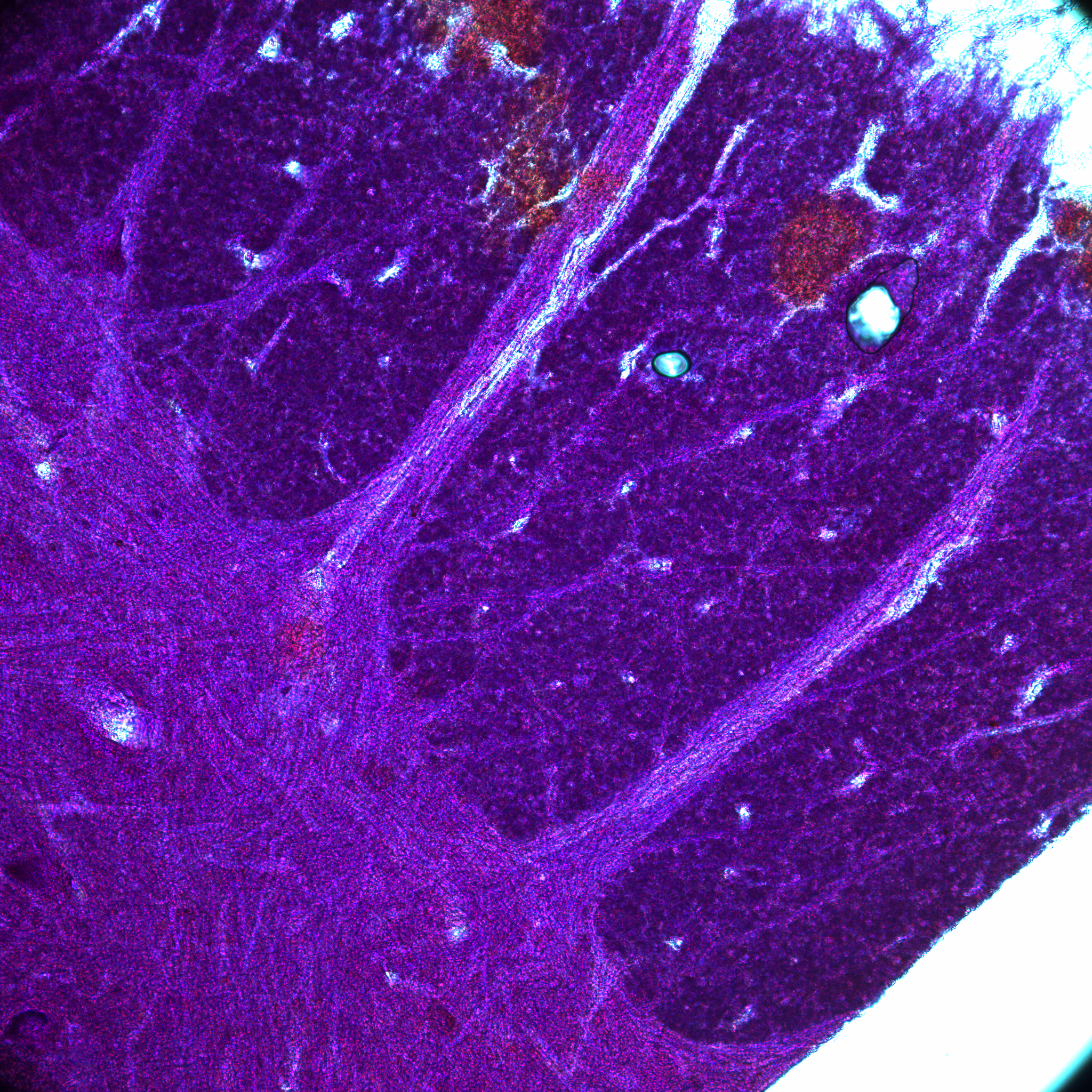

Supplement: Supplementary file 2 — Supplementary material [file mmc2.zip › data_sorted/human_sc/set2/Histology/Hum071409_HumanDTI_scans61-82_100X1_Nissl stained_50um thick_true color.tif]

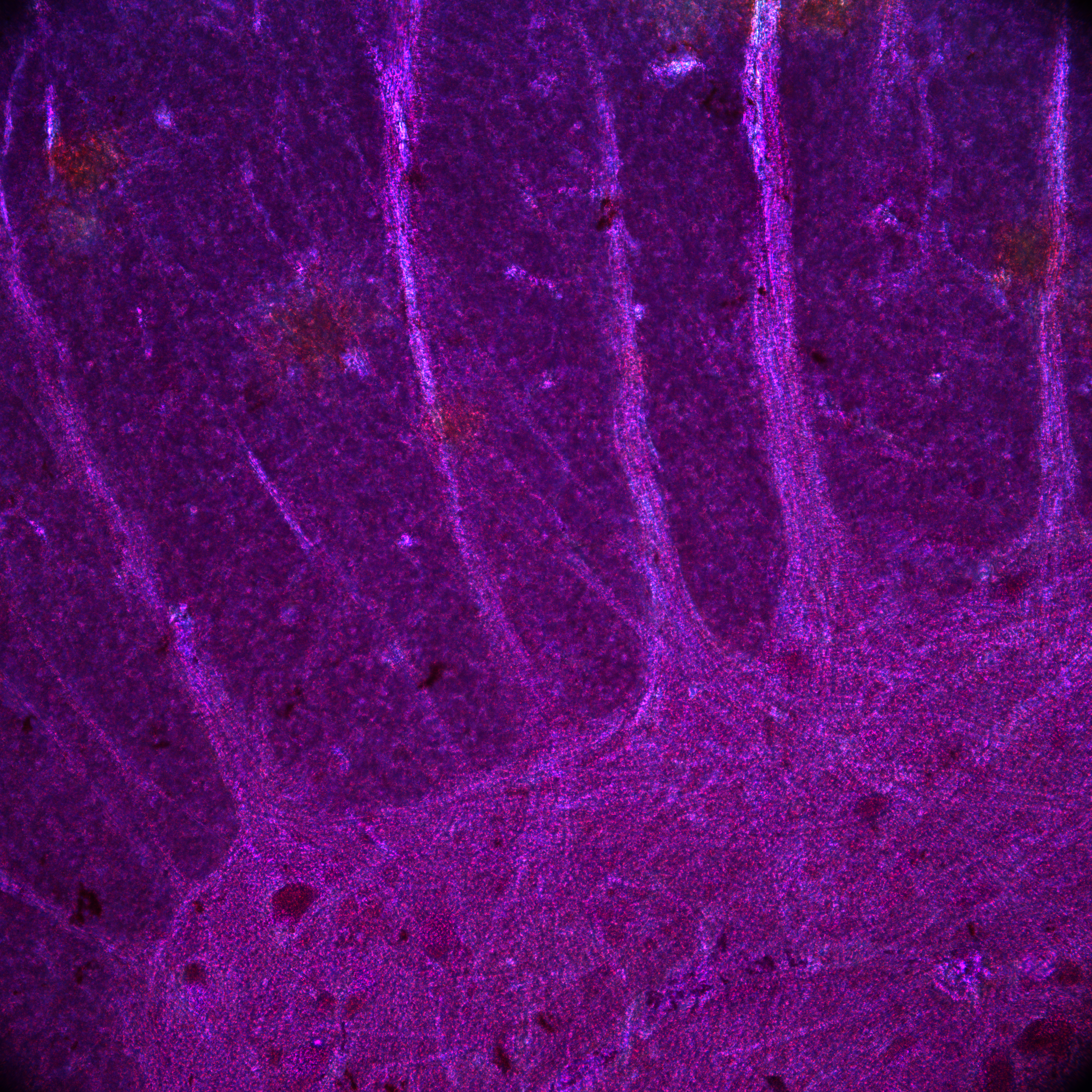

Supplement: Supplementary file 2 — Supplementary material [file mmc2.zip › data_sorted/human_sc/set3/Histology/Hum071409_HumanDTI_scans89-95_100X1_Nissl Stained_100um thick_false color.tif]

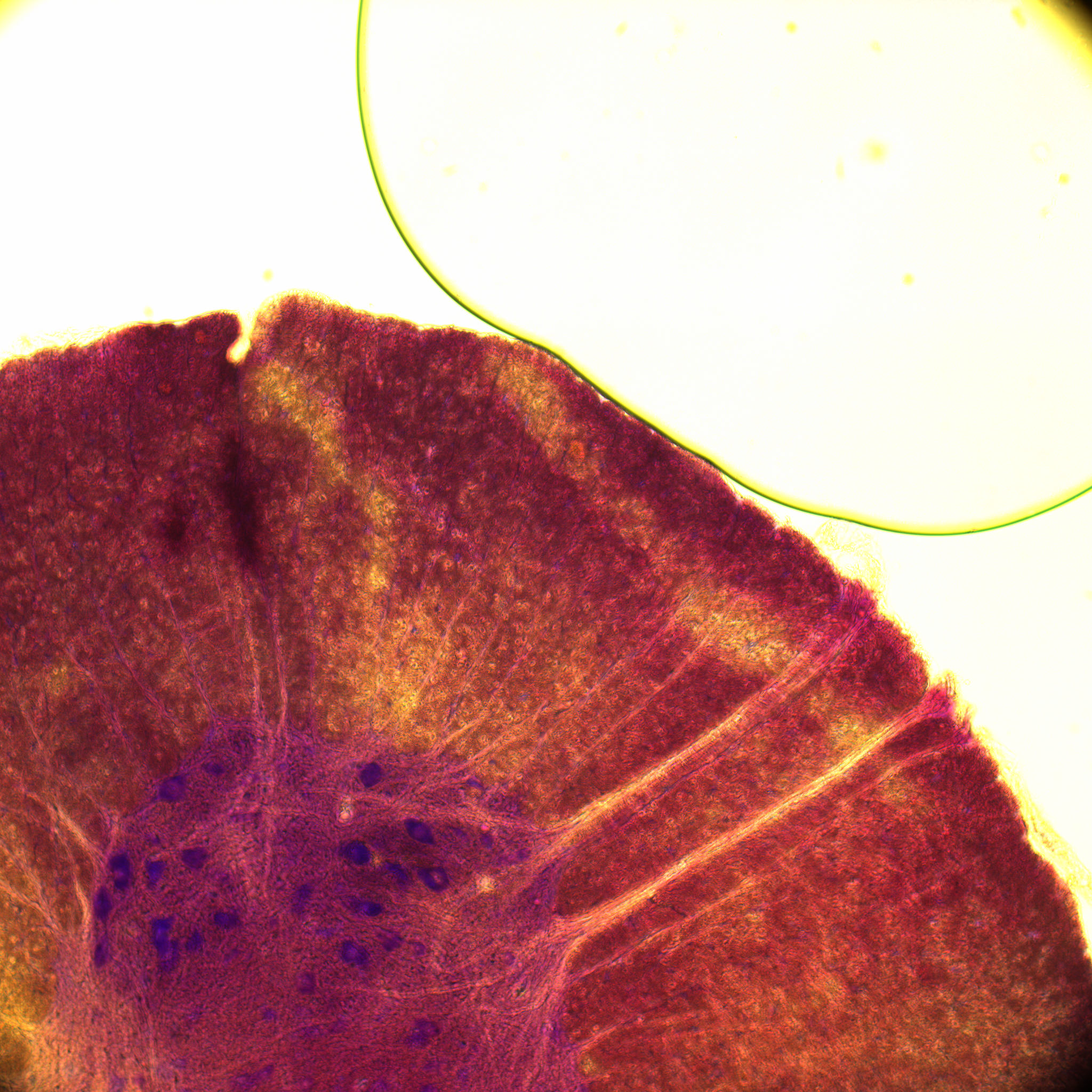

Supplement: Supplementary file 2 — Supplementary material [file mmc2.zip › data_sorted/rat_sc/set1/Histology/Mic072808_scans38 to 59_Nissl stain_cervical enlargement_50um thick_true color.tif]

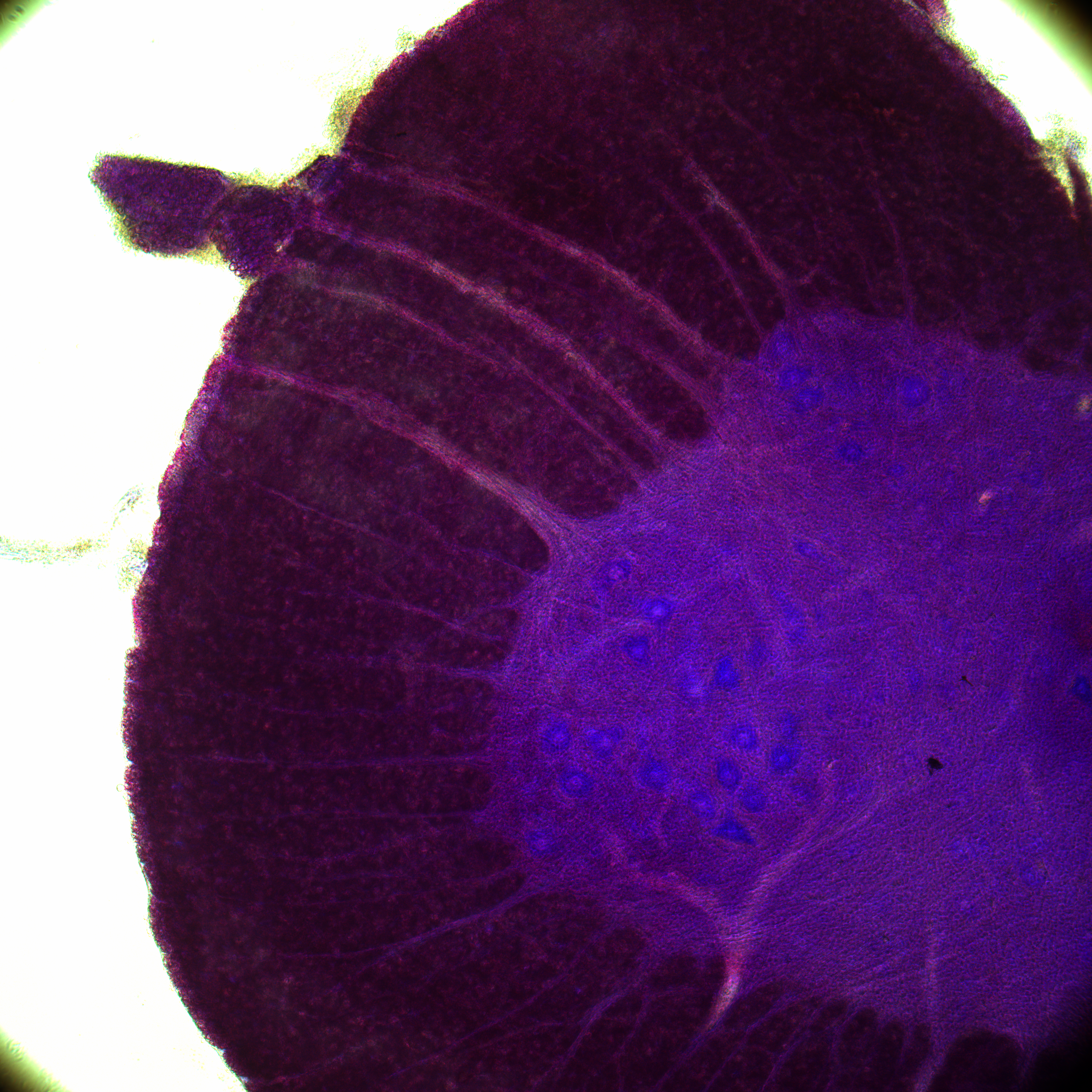

Supplement: Supplementary file 2 — Supplementary material [file mmc2.zip › data_sorted/rat_sc/set2/Histology/Mic072808_scans64 to 85_Nissl Stain_lumbar enlargement_50um thick_false color.tif]
